# A Case of Carcinoid Likely Causing Jejunal Intussusception

**DOI:** 10.1155/2014/949020

**Published:** 2014-03-16

**Authors:** Jennifer Matulich, Kelly Thurston, Dan Galvan, Subhasis Misra

**Affiliations:** Texas Tech University Health Sciences Center, 1400 South Coulter Street, Amarillo, TX 79106-1786, USA

## Abstract

A 34-year-old female was admitted to Emergency Department with an abrupt onset of diffuse abdominal pain. A CT scan done prior to her transfer revealed significant dilated loops of bowel as well as multiple target signs with likely torsed bowel. The patient consented to an exploratory laparotomy. During surgery, the proximal jejunum was found to be intussuscepted, a rare finding in an adult. There was evidence of mesenteric foreshortening throughout the small bowel and multiple whitish lesions within the mesentery, both consistent with the desmoplastic response that is characteristic of carcinoid. The interest for this case report comes from the patient's surgical findings of jejunal intussusception as well as her extensive history, which includes a bowel resection with an ileocolic anastomosis for presumed ischemia and a carcinoid tumor in the stomach which had been removed endoscopically.

## 1. Background

Carcinoid disease is known for its wide array of signs, symptoms, and ubiquitous gastrointestinal involvement. Carcinoid tumors are neuroendocrine in origin. The clinical presentation of the tumor depends on its location and most tumors are asymptomatic. In patients who are symptomatic, forty percent will present with abdominal pain. This may be due to intussusception, mesenteric ischemia, or a mechanical obstruction caused by the tumor [1]. The prevalence of carcinoid tumors is 1-2 cases per 100,000 persons, slightly more common in African Americans, with twenty-five to thirty-five percent arising in the small bowel (most commonly in the ileum, 91%) [2]. Carcinoid is positively associated with age, BMI, and postmenopausal women on hormone therapy [3]. Patients that present with symptoms of carcinoid syndrome, by definition, have metastasis of the tumor to the liver, and the prognosis for this select group of people is poor. Carcinoid syndrome occurs due to the release of secretory products such as serotonin, bradykinin, and histamine into the blood stream. The liver normally metabolizes these products, but this process is bypassed once it is involved. This causes signs similar to serotonin syndrome, including diarrhea, cutaneous flushing, and bronchospasm.

## 2. Case Presentation

A 34-year-old female was transferred from an outside hospital with a sudden onset of diffuse abdominal pain. Her history is significant for malrotation with subsequent volvulus, small bowel resection done for ischemic bowel, carcinoid tumor of the stomach, multiple gastric ulcers, and a tubular adenoma of the colon. She first presented in 2005 with suspected ischemic bowel, which was resected and repaired with an ileocolic anastomosis. Her next presentation was in January of 2013 at which time she had severe anemia with a hemoglobin of 4.2 mg/dL. An EGD revealed a carcinoid tumor in the lesser curvature of the stomach that was endoscopically removed. A polyp removed during colonoscopy was identified as a tubular adenoma. She presented again the next month and an abdominal CT revealed two lesions in the liver, a left adrenal nodule, and an umbilical hernia. The liver nodules prompted a workup carcinoid syndrome, but the urine was negative for 5-HIAA.

**Figure 1:**
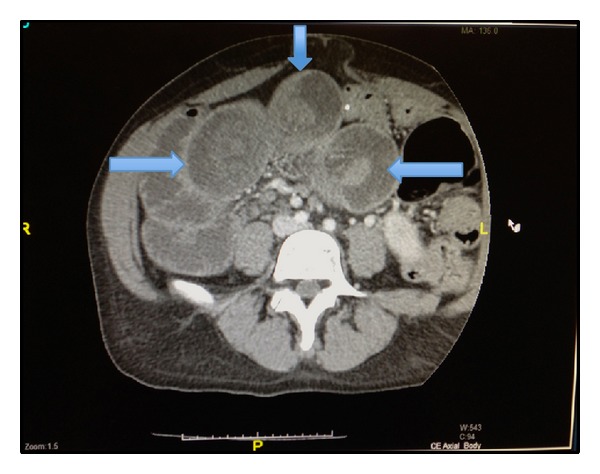
Abdominal CT scan with target signing of bowel (arrows).

**Figure 2:**
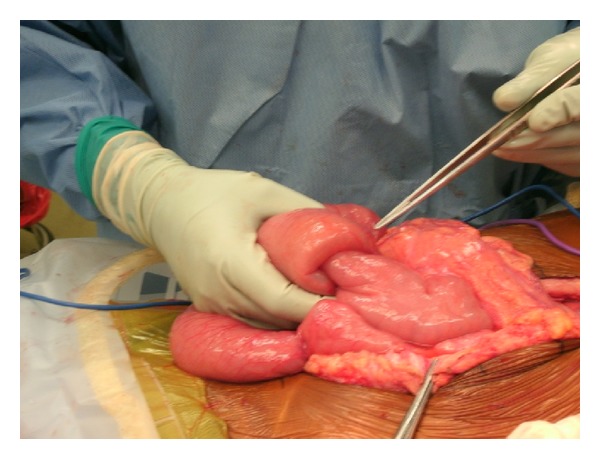
Intussuscepted bowel.

**Figure 3:**
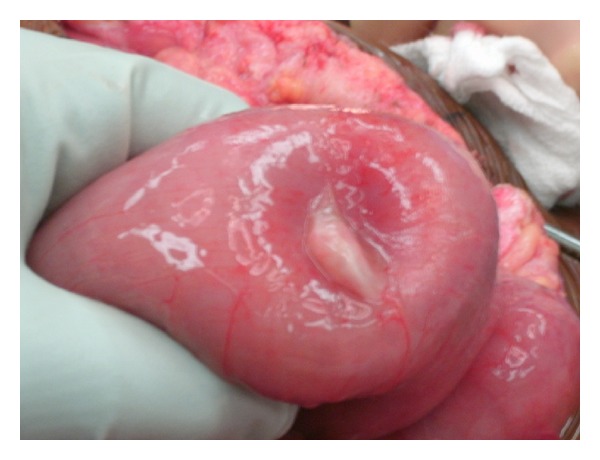
Suspected lead point of intussusception.

**Figure 4:**
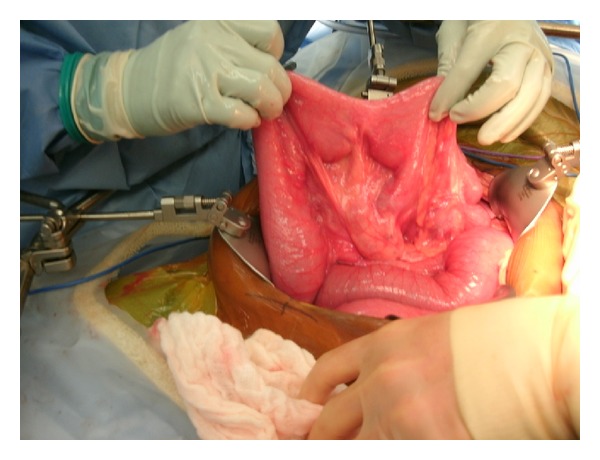
Area of narrowed bowel near the terminal ileum.

Upon her arrival to our hospital in August of 2013, she was noted to have moderate generalized tenderness on physical exam and her last bowel movement was earlier that day. An abdominal CT scan done prior to her arrival was suggestive of new marked mechanical traction of the distal small bowel. There were also three target signs present on imaging, a classic representation of intussuscepted bowel (Figure 1). The patient consequently underwent an exploratory laparotomy. The operation was initiated using a midline incision, and an area of intussusception was revealed in the proximal jejunum (Figure 2) and multiple fibrotic implants were present throughout the bowel and mesentery. The lead point of intussusception was identified (Figure 3). The compromised portion of bowel was resected and a jejunojejunal anastomosis was created. The bowel was then run from the Ligament of Treitz to the ileocecal anastomosis. An area of narrowed bowel was seen in the distal ileum near the site of the patient's previous ileocolic anastomosis (Figure 4). An ileoileal anastomosis was created to bypass this site given the presence of shortened bowel. In addition, there was mesenteric foreshortening throughout the small bowel, which is significant due to its tendency to cause twisting and obstruction of the bowel. An internal hernia and an umbilical hernia were also found but did not appear to be as lead points for the intussusception and were subsequently reduced.

## 3. Investigations

A CT scan was the primary tool for diagnosis in this patient. Multiple dilated loops of small bowel as well as target signs were present, indicating a possible obstruction as well as intussusception.

## 4. Differential Diagnosis

A patient that presents with diffuse abdominal pain may have a perforated viscus, a bowel obstruction, or some other process that has resulted in irritation of the peritoneum. The most common cause of a bowel obstruction is adhesions from previous abdominal surgery. This was initially suspected in this patient given her surgical history. A thorough history and physical including onset and location of pain may help to rule out various causes of an acute abdomen. While often times the management and treatment of these conditions are the same, it is important to properly investigate the etiology of disease to avoid missing potential sequelae. When diagnosing a patient with potential carcinoid, it is important to remember that it may be a clinical diagnosis based on the appearance and location of the tumors throughout the GI tract, in addition to mesenteric foreshortening. In the presence of a bowel obstruction, the etiology often cannot be established prior to an exploratory laparotomy. The most common causes of bowel obstruction are adhesions and hernias, but in our case neither was determined to be the culprit ultimately.

## 5. Treatment

In most cases of focal carcinoid disease, surgical resection is both diagnostic and curative. However, the patient is often asymptomatic and does not present clinically until the disease has caused an obstruction of the bowel or manifestations of carcinoid syndrome indicate metastasis to the liver. The patient in our case study appeared to have multiple manifestations of disease and chronic anemia that may have been linked to her diffuse gastrointestinal involvement.

## 6. Outcome and Follow-Up

The patient was discharged on postoperative day eight and was followed up as an outpatient in the clinic. She had no apparent complications from the operation and appeared to recover well. It is recommended that the patient follow up every 6 months.

## 7. Discussion

A patient that presents with diffuse abdominal pain may have a perforated viscus, a bowel obstruction, or some other process that has resulted in irritation of the peritoneum. The most common cause of a bowel obstruction is adhesions from previous abdominal surgery. This was initially suspected in this patient given her surgical history. A thorough history and physical including onset and location of pain may help to rule out various causes of an acute abdomen. While often times the management and treatment of these conditions are the same, it is important to properly investigate the etiology of disease to avoid missing potential sequelae. When diagnosing a patient with potential carcinoid, it is important to remember that it may be a clinical diagnosis based on the appearance and location of the tumors throughout the GI tract, in addition to mesenteric foreshortening. In the presence of a bowel obstruction, the etiology often cannot be established prior to an exploratory laparotomy. The most common causes of bowel obstruction are adhesions and hernias, but in our case neither was determined to be the culprit ultimately. Carcinoid tumors have a variety of clinical presentations, depending on their location and size. Tumors in the small bowel are hypothesized to arise from subepithelial endocrine cells and are most commonly located within sixty centimeters of the ileocecal valve. Abdominal pain is the clinical presentation in forty percent of patients and it may be linked to a mechanical bowel obstruction from the tumor itself or intussusception. Mesenteric ischemia caused by local fibrosis is also seen and is representative of the desmoplastic response that is pathognomonic for carcinoid [1]. Other gastrointestinal tumors such as a GIST or a leiomyoma lack this fibrous tissue reaction. In addition, small bowel adenocarcinoma is associated with a limited dense reaction in the mesentery. Metastatic melanoma can characteristically present with target lesions on contrast studies much like intussuscepted bowel, so this must be ruled out as well [4]. Intussusception is a rare cause of intestinal obstruction in adults [5]. This occurrence accounts for about five percent of all intussusceptions and one percent of bowel obstructions [6]. There is limited data on jejunal intussusception, but the studied cases have revealed a tumor or some other intestinal abnormality as the causal agent in the majority of cases [7]. Therefore, the presence of intussusception in an adult is a significant finding, as its presence may allude to an underlying malignancy. It follows that resection without reduction is a necessity in most cases in order to abrogate the origin of the intussusception [8]. Fibrosis is a classic feature found in patients with neuroendocrine tumors arising in the ileum and jejunum [9]. This process can occur in the mesentery as a desmoplastic response and a bowel obstruction is likely to ensue [10]. The patient in this case study had several hallmark findings associated with carcinoid tumors. Her extensive medical history illustrates the unique findings of this disease in the gastrointestinal tract including jejunal intussusception and thus was the basis for our study.

## Learning Points

1. Jejunal intussusception is rare in both adults and children.
2. Risk factors for carcinoid include family history of MEN I, African American race, tobacco use, atrophic gastritis, Zollinger-Ellison syndrome, and pernicious anemia.
3. There is no standardized screening for carcinoid.
4. Mesenteric foreshortening is the hallmark of carcinoid in the gastrointestinal system.
5. Target sign on CT scan is indicative of intussusception and the need for an exploratory laparotomy.
6. Intussusception in an adult warrants surgery to evaluate the underlying cause.
